# G12 Signaling through c-Jun NH_2_-Terminal Kinase Promotes Breast Cancer Cell Invasion

**DOI:** 10.1371/journal.pone.0026085

**Published:** 2011-11-07

**Authors:** Juhi Juneja, Ian Cushman, Patrick J. Casey

**Affiliations:** 1 Department of Pharmacology and Cancer Biology, Duke University Medical Center, Durham, North Carolina, United States of America; 2 Program in Cancer and Stem Cell Biology, Duke-NUS Medical School, Singapore, Republic of Singapore; Sun Yat-sen University Medical School, China

## Abstract

Signaling through the heterotrimeric G protein, G12, via Rho induces a striking increase in breast cancer cell invasion. In this study, evidence is provided that the c-Jun NH_2_-terminal kinase (JNK) is a key downstream effector of G12 on this pathway. Expression of constitutively-active Gα12 or activation of G12 signaling by thrombin leads to increased JNK and c-Jun phosphorylation. Pharmacologic inhibition of JNK or knockdown of JNK expression by siRNA significantly decreases G12-induced JNK activation as well as the ability of breast cancer cells to invade a reconstituted basement membrane. Furthermore, expression of dominant-negative Rho or treatment of cells with an inhibitor of the Rho kinase, ROCK, reduces G12-induced JNK and c-Jun activation, and ROCK inhibitor treatment also inhibits G12-induced cellular invasion. JNK knockdown or ROCK inhibitor treatment has no effect on activation of Rho by G12. Taken together, our data indicate that JNK activation is required for G12-induced invasion of breast cancer cells and that JNK is downstream of Rho and ROCK on this pathway. This study implicates a G12-stimulated mitogen-activated protein kinase cascade in cancer cell invasion, and supports a role for JNK in cancer progression.

## Introduction

Heterotrimeric guanine nucleotide-binding regulatory proteins (G proteins) transduce extracellular signals from heptahelical G protein-coupled receptors to intracellular effectors, thus mediating numerous cellular responses [1], [2]. G proteins are classified into four subfamilies based on the sequence identity of their alpha subunits: Gs, Gi, Gq and G12. The G12 subfamily members, Gα12 and Gα13, are the most potent transforming alpha subunits known thus far [3], [4]. In fact, Gα12 was identified as the transforming oncogene in a study using an expression cloning method to identify putative oncogenes of soft tissue sarcoma [5]. Subsequent work has implicated Gα12 and Gα13 in various physiological and pathophysiological processes such as cell growth and proliferation, cytoskeleton rearrangement, regulation of cell polarity, cellular adhesion, migration and metastasis [6]–[9].

Activation of G12 proteins impacts on several signaling pathways including mitogen-activated protein kinase (MAPK) cascades, where they can coordinate the stimulation and/or inhibition of MAPKs [10]–[12]. G12-induced regulation of MAPK signaling pathways appears to be primarily mediated by the Rho family of monomeric GTPases, which are the most extensively characterized downstream effectors of G12 proteins [3], [12]. Gα12 and Gα13 stimulate JNK activity [12]–[14]; a major outcome of JNK activation in cells is the phosphorylation of the transcription factor c-Jun, leading to increased transcription of genes involved in proliferation and survival, cell motility and invasion, among others [15]. Thus, some of the biological consequences of Gα12/13 activation could potentially be due to an impact on c-Jun activity [16]–[18].

We recently reported a role for the G12 subfamily of G proteins in cancer invasion and metastasis [19], [20]. Stimulation of G12 signaling induced invasion of breast cancer cells *in vitro* in a Rho-dependent manner, and inhibition of G12 signaling significantly reduced metastatic dissemination of breast cancer cells *in vivo* [19]. In the current study, we sought to examine if JNK was a downstream effector on the pathway stimulated by G12 that leads to breast cancer cell invasion. As expected, G12 signaling increased JNK phosphorylation in breast cancer cells. Importantly, we found that activation of JNK is required for breast cancer cell invasion induced by activated Gα12, indicating that JNK is an important downstream mediator of activated G12 in these cells. These findings have implications for understanding the molecular events contributing to metastatic dissemination of tumor cells.

## Materials and Methods

### Cell lines and reagents

The MDA-MB-231 cell line (obtained from American Type Culture Collection) and the 4T1 cell line (a generous gift from Fred Miller, Barbara Ann Karmanos Cancer Center, Detroit) [19] were maintained in DMEM (Mediatech Inc, Manassas, VA) supplemented with 10% fetal bovine serum (Atlas Biologicals, Fort Collins, CO). The JNK 1/2 siRNA [21] was synthesized by Integrated DNA Technologies, Inc. (Coralville, IA). Oligofectamine reagent was from Invitrogen (Carlsbad, CA). The JNK inhibitor SP600125 and ROCK inhibitor Y-27632 were purchased from Calbiochem (San Diego, CA). Recombinant human thrombin was from Enzyme Research Laboratories (South Bend, IN). Growth-factor reduced Matrigel was from BD Biosciences (Bedford, MA) and fibronectin from human plasma was from Sigma (St. Louis, Mo). cDNAs encoding Gα12QL, HA-RGS2 (both from University of Missouri cDNA Resource Center, Rolla, MO) and myc-p115RGS (a gift of Tohru Kozasa, University of Illinois, Chicago) were used to create recombinant adenoviruses as described previously [19]. Dominant-negative RhoA adenovirus was a gift from Terete Borrás (University of North Carolina, Chapel Hill). The pGEX-2T plasmid containing a GST fusion to the rhotekin RhoA-binding domain (GST-RBD) was kindly provided by Robert Lefkowitz (this institution). Phospho-SAPK/JNK (Thr^183^/Thr^185^) antibody was from Cell Signaling Technology, Inc (Beverly, MA). Antibodies to Gα12, phospho-c-Jun, JNK, RhoA, α-tubulin were purchased from Santa Cruz Biotechnology (Santa Cruz, CA). Anti-c-myc was from Zymed Laboratories, (San Francisco, CA) and anti-HA was from Roche Applied Science (Indianapolis, IN). Protease inhibitors were purchased from Sigma and Roche Applied Science.

### JNK activation assay

MDA-MB-231 cells were seeded at a density of 200,000 per well in 6-well plates and infected with the indicated adenovirus for ∼6 h. For JNK knockdown experiments, cells were seeded at 100,000 per well and transfected with siRNA for 70–72 h before infection. 260 pmol (20 pmol/µl) of control or JNK siRNA was transfected per well using Oligofectamine reagent. Cells were starved for ∼2–20 h before lysis in JNK activation buffer [22]. 4T1 cells were infected with the indicated adenovirus for 18–19 h and starved for ∼9 h before lysis. For inhibiting JNK or ROCK, cells were treated with their respective inhibitors, 20 µM SP600125 or 10 µM Y-27632, for 2 h before lysis. Cells were lysed for 10 min and lysates prepared by centrifugation at 16000×g for 5 min at 4°C. Equal amounts of total protein from lysates were separated by SDS-PAGE and subjected to immunoblot analysis to detect levels of phospho-JNK and phospho-c-Jun.

### Cell invasion assay

For invasion assays, transwell chambers (8 µm pore size, polycarbonate filters, 6.5 mm diameter; Costar) were coated with 50 µg growth-factor-reduced Matrigel [19]. MDA-MB-231 cells that had been starved for 18–20 h, or 4T1 cells that had been starved for 8 h, were suspended in starvation media (DMEM containing 0.1% BSA), and 4×10^4^ cells were plated on top of the Matrigel in the upper chamber with or without agonists as indicated. Cells were allowed to invade Matrigel towards 5 µg/ml fibronectin in starvation media or towards 3% FBS. After 24 h, cells in the upper chamber that did not invade were removed by swiping with cotton swabs. The transwell membranes were then stained using the Hema3 staining kit (Fisher Scientific) and cells on the underside of the membranes that had invaded the Matrigel were counted under a Nikon TS100 microscope. Four high-powered fields were counted for each membrane.

### Rho activation assay

Cells were seeded at a density of 200,000 (or 100,000 for siRNA transfections) per well in 6-well plates. Cells were infected with the indicated adenovirus for 6–7 h, and starved for 18 h before lysis. Rho activation assays were performed as described previously [22]. Rho-GTP in cell lysates was precipitated using the activated Rho-binding domain of rhotekin and detected by separation on SDS-PAGE followed by immunoblot analysis.

### Rhodamine-phalloidin staining

MDA-MB-231 cells were plated on coverslips. After starvation for ∼18 h, cells were washed in PBS, fixed in 4% paraformaldehyde and permeabilized in 0.1% Triton X-100. Cells were blocked in 5% serum and incubated with rhodamine-phalloidin for 1 h at room temperature. The coverslips were mounted on glass slides and images acquired on a Zeiss Axio Imager fluorescence microscope using the MetaMorph 7.5 software. Fluorescence intensities were quantified using the MetaMorph 7.5 software.

### Statistical analysis

Data were analyzed using GraphPad Prism v4 (GraphPad Software Inc.). Data are presented as mean ± SEM and compared by one-way ANOVA, followed by Tukey's multiple comparison test to obtain P values. A probability of *p*<0.05 was considered significant.

## Results

### G12 signaling activates JNK and promotes breast cancer cell invasion

**Figure 1:**
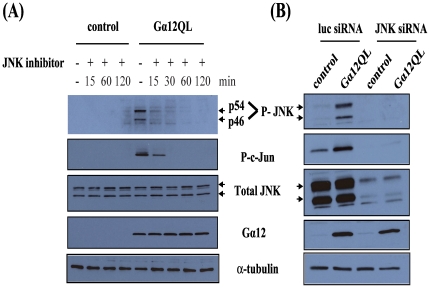
Gα12QL activates JNK in breast cancer cells. MDA-MB-231 cells were infected with GFP (control) or Gα12QL adenovirus for 6–7 h. (A) Cells were starved for 2 h and then treated for the indicated time with JNK inhibitor SP600125 (20 µM) prior to lysis in JNK activation buffer and processing for analysis (see *Materials and Methods*). (B) Cells were transfected with JNK siRNA or control (luc) siRNA for ∼72 h prior to infection with adenovirus as in (A). Cells were starved for 18 h before being lysed. For both panels, equal amounts of total protein in lysates were subjected to SDS-PAGE and immunoblot analysis to assess levels of phospho-JNK (P-JNK, p54 and p46) and phospho-c-Jun (P-c-Jun). Total levels of JNK and Gα12 expression, along with α-tubulin as a loading control, are also shown. Each panel shows the results of a single experiment that is representative of three or more independent experiments.

As we previously reported, ectopic expression of activated Gα12 (Gα12QL) in MDA-MB-231 breast cancer cells leads to the activation of JNK [22]; see also Figure 1A. Here, we studied this process both by examining c-Jun phosphorylation as well as JNK phosphorylation to confirm an increase in JNK activity. The JNK protein is encoded by three genes (*jnk1*, *jnk2* and *jnk3*) [23]. JNK1 and JNK2 proteins are ubiquitously expressed while JNK3 expression is restricted to brain, heart and testis. Each JNK is expressed as a short form (46 kDa) and a long form (54 kDa); both forms are detected by the phospho-JNK antibody used in this study. As shown in Figure 1A, expression of Gα12QL induces JNK phosphorylation (lane 5) and this could be rapidly and efficiently blocked by treating cells with the pharmacologic inhibitor of JNK phosphorylation, SP600125. Since it has been reported that SP600125 may not be a specific inhibitor of JNK activation [24], we also employed siRNA-mediated silencing of JNK expression. JNK knockdown of >90% was accompanied by a complete loss of Gα12QL-stimulated c-Jun phosphorylation in MDA-MB-231 cells (Figure 1B), but did not affect proliferation or viability of MDA-MB-231 cells as assessed by an MTS assay (data not shown). Hence, in addition to an impact on JNK activation, expression of Gα12QL induces elevated levels of c-Jun phosphorylation, and c-Jun activation resulting from the introduction of activated Gα12 is blocked both by pharmacologic and siRNA-mediated inhibition of JNK activation (Figures 1A, B).

**Figure 2:**
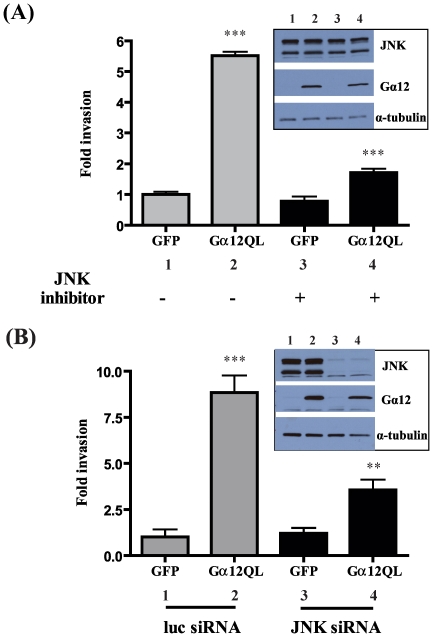
Gα12QL promotes cell invasion through activation of JNK. MDA-MB-231 cells were infected with GFP (control) or Gα12QL adenovirus for 6–7 h and starved for ∼20 h. (A) Cells were pre-treated for 2 h with JNK inhibitor SP600125 (20 µM) and allowed to invade Matrigel for 24 h, in the presence of the inhibitor or vehicle control, in a transwell invasion assay (see *Materials and Methods*). (B) Cells were transfected with JNK siRNA or control (luc) siRNA for ∼72 h before being infected with adenovirus. Cells expressing Gα12QL or GFP were allowed to invade Matrigel for 24 h. For both panels, the invasion assay was set up towards 5 µg/ml fibronectin. Cells that invaded were stained and counted in four random optical fields for each transwell, with three transwells per condition. Results are expressed as fold change in invasion compared to vehicle-treated GFP control cells. The *insets* show immunoblot analysis of the levels of Gα12, JNK and α-tubulin (loading control). Data is presented as mean ± SE from a single experiment that is representative of three independent experiments. ** *p*<0.01 and *** *p*<0.001, as determined by one-way ANOVA, followed by Tukey's multiple comparison test to obtain *p* values.

JNK activation has been implicated in promoting invasion of transformed fibroblasts and of several cancer cell lines [25]–[28]. Since expression of Gα12QL in MDA-MB-231 cells induces their invasion *in vitro* [19], we sought to determine if JNK might be a downstream effector of G12 signaling leading to invasion of breast cancer cells. Indeed, expression of Gα12QL in MDA-MB-231 cells induced an approximately six-fold increase in cell invasion through Matrigel, and inhibition of JNK activation by SP600125 resulted in a nearly 70% decrease in Gα12QL-stimulated cell invasion (Figure 2A). Furthermore, while Gα12QL induced an eight-fold increase in invasion of control cells, blocking JNK expression by siRNA-mediated knockdown caused a significant (50–60%) decrease in their ability to invade Matrigel in response to introduction of activated Gα12 (Figure 2B). There was no effect of silencing JNK expression on the invasion of control cells, demonstrating that this effect is specific to Gα12QL-induced invasion. These data strongly implicate JNK activation as being important in breast cancer cell invasion induced by activated Gα12.

### G12 activates a ROCK to JNK signaling axis in breast cancer cells

**Figure 3:**
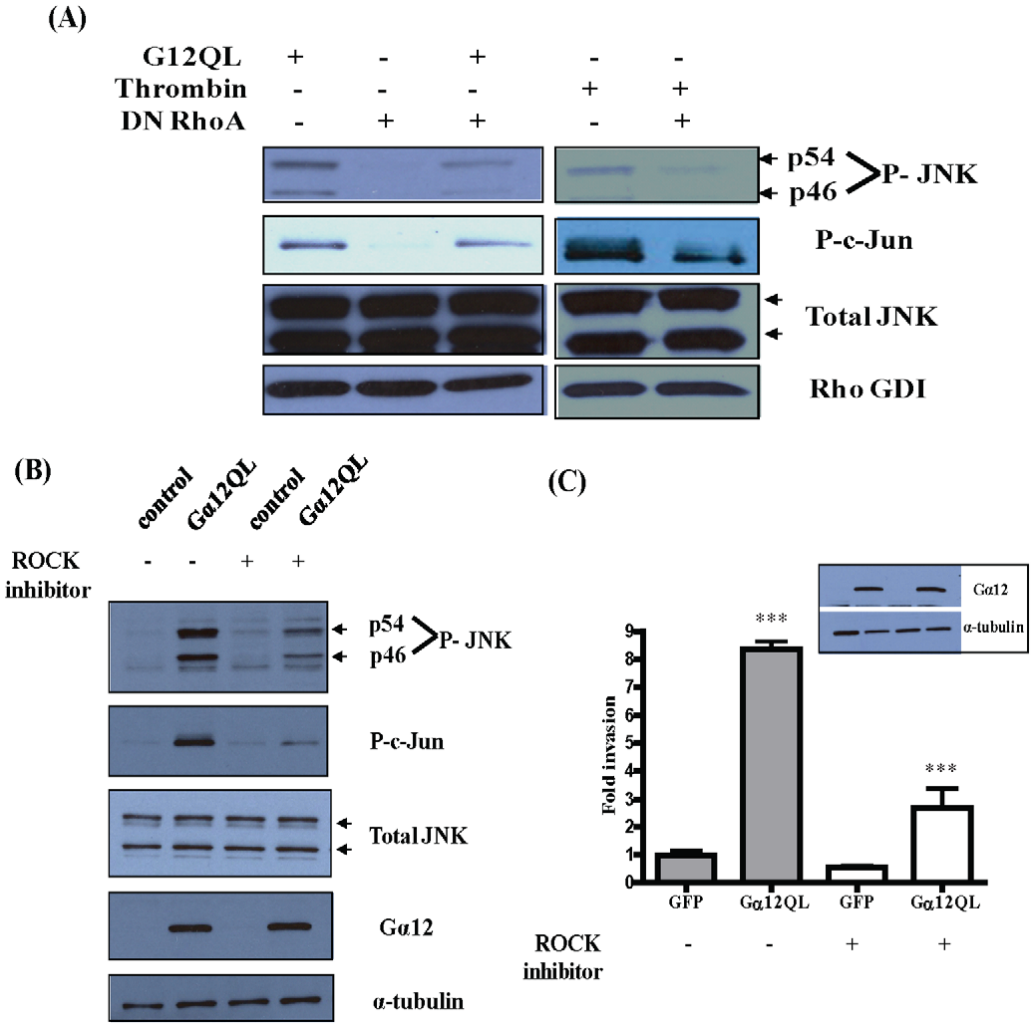
Gα12QL activates a ROCK-JNK signaling axis. MDA-MB-231 cells were infected with adenovirus harboring GFP (control), Gα12QL or dominant-negative RhoA (DN RhoA) for 6–7 h. (A) Cells were starved and treated with 1 U/ml thrombin for 10 min, as indicated, before lysis in JNK activation buffer. Equal amounts of total protein in lysates were subjected to SDS-PAGE and immunoblot analysis to assess levels of phospho-JNK (P-JNK, p54 and p46) and phospho-c-Jun (P-c-Jun). Total level of JNK expression, along with Rho GDI as a loading control, is also shown. (B) Cells were starved and treated with ROCK inhibitor Y-27632 (10 µM) for 2 h before lysis in JNK activation buffer. Equal amounts of total protein in lysates were subjected to SDS-PAGE and immunoblot analysis to assess levels of phospho-JNK (P-JNK, p54 and p46) and phospho-c-Jun (P-c-Jun). Total levels of JNK and Gα12 expression, along with α-tubulin as a loading control, are also shown. Data shown in (A) and (B) are from a single experiment that is representative of three independent experiments. (C) Cells infected with GFP or Gα12QL adenovirus were starved for ∼18 h, then pre-treated with Y-27632 (10 µM) for 2 h and allowed to invade Matrigel for 24 h in the presence of the inhibitor, towards 5 µg/ml fibronectin. Cells that invaded were stained and counted in four random optical fields for each transwell, with three transwells per condition. Results are expressed as fold change in invasion compared to vehicle-treated GFP control cells. The *inset* shows immunoblot analysis of Gα12 and α-tubulin (loading control) levels. Data is presented as mean ± SE from a single experiment that is representative of three independent experiments. *** *p*<0.001, as determined by one-way ANOVA, followed by Tukey's multiple comparison test to obtain *p* values.

Since activation of Rho is required for G12 to induce invasion in breast cancer cells [19], we examined the possibility that JNK is downstream of Rho on this signaling pathway. Expression of dominant-negative RhoA (DN RhoA) in MDA-MB-231 cells significantly inhibited Gα12QL-indued JNK as well as c-Jun phosphorylation (Figure 3A), indicating that JNK is indeed activated downstream of Rho. Furthermore, stimulation of endogenous G12 signaling by thrombin, which activates the G12-coupled protease-activated receptor (PAR-1) [29], led to JNK and c-Jun activation in breast cancer cells in a manner that was inhibited by expression of DN RhoA (Figure 3A).

The best characterized effector of activated Rho is the Rho kinase, ROCK, which controls actin polymerization and leads to serum response factor (SRF) activation [30], [31]. Additionally, previous studies have shown that Rho can activate JNK through ROCK [32]–[34]. When treated with the ROCK inhibitor Y-27632, MDA-MB-231 cells expressing Gα12QL show a significant decrease in the levels of JNK and c-Jun phosphorylation as compared to untreated cells (Figure 3B). In addition, treatment of cells with the ROCK inhibitor decreases G12-induced cell invasion by about 70% (Figure 3C).

**Figure 4:**
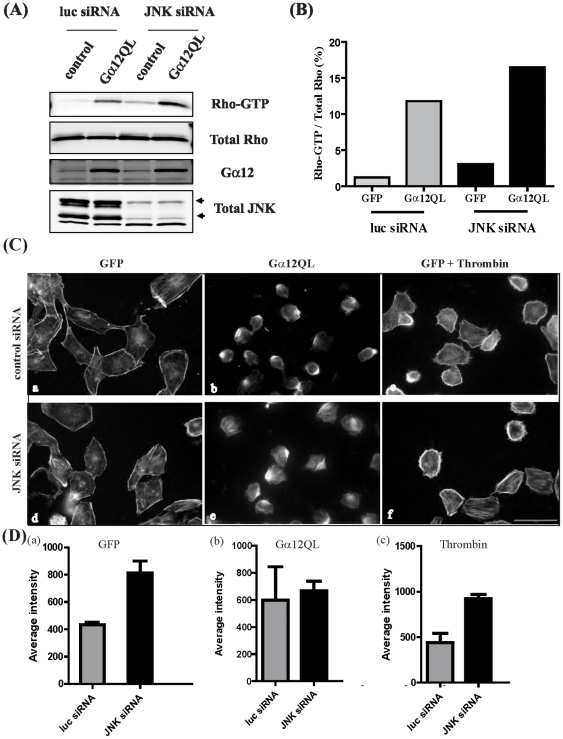
ROCK and JNK signal downstream of Gα12QL-activated Rho. (A) MDA-MB-231 cells were transfected with control (luc) siRNA or JNK siRNA for 3 days prior to infection with GFP (control) or Gα12QL adenovirus for 6–7 h. Cells were starved for ∼18 h and lysed. Lysates were subjected to pull-down assays using a GST fusion construct of the activated RhoA-binding domain of rhotekin, and levels of precipitated Rho-GTP were determined by immunoblot analysis using anti-RhoA antibody. Total levels of RhoA, Gα12 and JNK in the lysates are also shown. (B) The data in the anti-Rho immunoblots shown in (A) was quantified by plotting the intensities as percentage of activated Rho to total Rho for each sample. (C) MDA-MB-231 cells were plated on coverslips and transfected with siRNA as stated above, prior to infection with GFP (control) (*panels a*, *d*) or Gα12QL (*panels b*, *e*) adenovirus. Cells were starved for ∼18 h, and those in two conditions (*panels c*, *f*) treated with 1 U/ml thrombin for 10 min as indicated before all cells were fixed and stained with rhodamine-phalloidin to visualize actin (see *Materials and Methods*). Representative images for each condition are shown. Bar, 50 µM. Each panel shows the results of a single experiment that is representative of three independent experiments. (D) Fluorescence intensities of rhodamine-phalloidin staining were quantified using MetaMorph 7.5 software. Average fluorescence intensities are plotted as mean ± SE for data from two slides imaged with the same exposure time for every condition shown; ∼220 cells (*panels a*, *c*), and ∼120 cells (*panel b*) quantified per slide.

Treatment of cells with the ROCK inhibitor did not affect Gα12QL-stimulated Rho activation (data not shown). To assess whether Rho activation by G12 was affected by JNK inhibition, i.e. whether Rho is upstream of ROCK and JNK as would be expected, we compared the levels of active Rho in lysates from cells in which JNK was inhibited either pharmacologically or by RNAi-mediated suppression. Comparison of Rho-GTP levels in cells expressing Gα12QL that had been transfected with control or JNK siRNA clearly demonstrated that JNK knockdown did not affect activation of Rho by Gα12QL (Figure 4A). Taken together, these data suggest that JNK is activated downstream of the Rho-ROCK axis in the signaling pathway triggered by G12 that promotes invasion of breast cancer cells. Interestingly, Gα12QL-stimulated Rho-GTP levels appear to be higher in cells in which JNK expression has been knocked down by siRNA compared to that in control cells (Figure 4A). When Rho-GTP levels were quantified and analyzed as a percentage of activated Rho to total Rho in each sample (Figure 4B), silencing JNK expression was indeed determined to elicit a significant increase in Rho activation status. This finding suggests the possible existence of a feedback signaling pathway in which reduction of JNK levels leads to increased Rho activation.

Additional evidence in support of a JNK-Rho feedback mechanism was provided by the finding that rhodamine-phalloidin staining of the actin cytoskeleton was increased in cells in which JNK expression was reduced by siRNA treatment (Figure 4C, D). It is well-documented that activation of Rho leads to stress fiber formation in several cell types [35]. Further, G12 proteins have been shown to stimulate stress fiber formation in a Rho-dependent manner in fibroblasts [36]. Under normal culture conditions, MDA-MB-231 breast cancer cells exhibit few or no stress fibers (Figure 4C, panel a). Gα12QL expression induced stress fiber assembly in these cells as is evident from the intense rhodamine-phalloidin staining compared to control cells (Figure 4C, panel b). As we previously reported [22], expression of Gα12QL in MDA-MB-231 breast cancer cells also caused a Rho-dependent change from a flattened to a rounded morphology (Figure 4C, panel b). Interestingly, there appears to be a distinct increase in stress fiber formation in Gα12QL-expressing as well as control (GFP) cells transfected with JNK siRNA (Figure 4C, panels a, b, d, e).

Similar to the expression of activated Gα12 in MDA-MB-231 cells, receptor-mediated stimulation of G12 signaling by treatment with thrombin caused a reorganization of the actin cytoskeleton and stress fiber formation compared to untreated cells, and an increase in stress fiber formation when JNK expression is knocked down (Figure 4C, panels c, f). Quantification of the fluorescence intensities of rhodamine-phalloidin staining in control versus JNK siRNA cells clearly shows that stress fiber formation is increased when JNK expression is knocked down (Figure 4D, panel a). Although Gα12QL-induced stress fiber formation is also increased in cells treated with JNK siRNA, the difference is only marginal compared to control cells, likely because of strong and persistent stimulation of Rho-mediated signaling by activated Gα12 even in control cells (Figure 4D, panel b). Thrombin-treated cells in which JNK expression is knocked down, however, showed a significant increase in rhodamine-phalloidin staining compared to control cells (Figure 4D, panel c). These findings reinforce the notion of the existence of a feedback pathway from JNK back to Rho, such that reduction of JNK expression leads to increased Rho activation and therefore increased stress fiber assembly.

### Thrombin signals via G12 to activate JNK in breast cancer cells, and JNK is required for stimulated 4T1 cell invasion

We next sought to assess whether Gα12-induced phosphorylation of JNK occurred during receptor-mediated activation of G12 proteins, and whether JNK has a role in receptor-stimulated breast cancer cell invasion. When 4T1 mouse mammary carcinoma cells stably expressing p115RGS (regulator of G protein signaling domain of p115RhoGEF, a construct used to block G12 signaling) were implanted in the mammary fat pads of female mice, there was a striking decrease in metastasis and increase in metastasis-free survival of these mice compared to control mice bearing tumors in which G12 signaling had not been blocked, suggesting that G12 promotes metastasis in mice by stimulating invasion from the primary tumor [19]. Hence, we decided to examine the effect of JNK activation on thrombin-mediated invasion in the 4T1 cells, since we had relevant *in vivo* data for a critical role of G12 proteins in metastatic progression of these cells.

**Figure 5:**
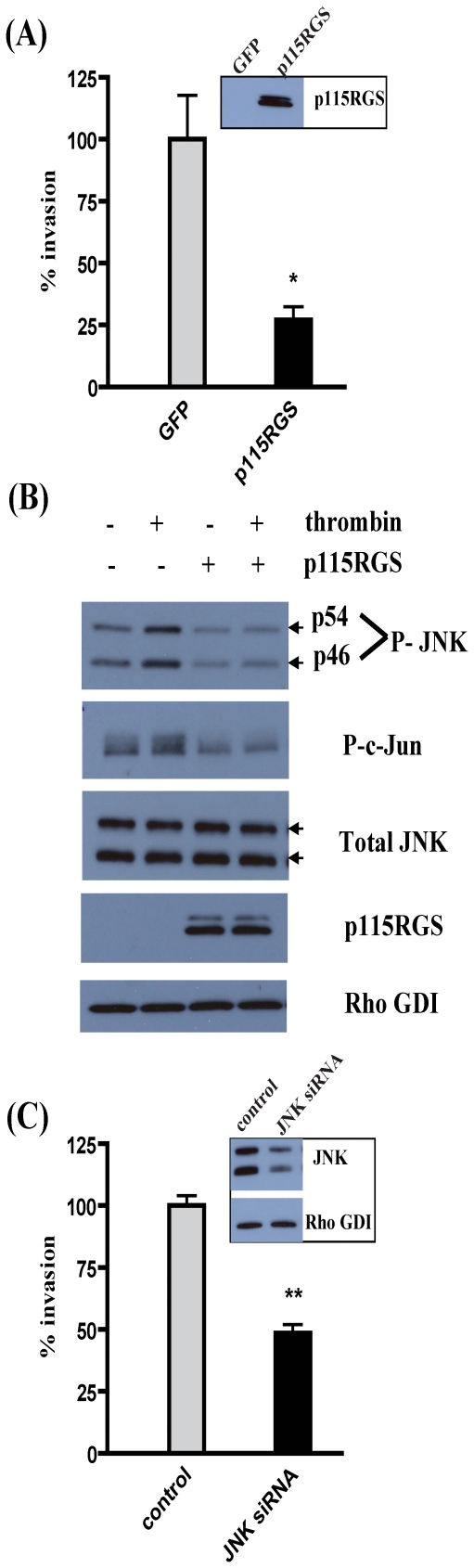
JNK is activated downstream of G12 signaling in 4T1 mammary carcinoma cells and is required for cell invasion. (A) 4T1 cells were infected with adenovirus harboring GFP (control) or the RGS domain of p115RhoGEF (p115RGS) for ∼20 h, and then starved for 8 h. Cells were allowed to invade Matrigel for 24 h in the presence of thrombin (1 U/ml), towards 3% FBS. Cells that invaded were stained and counted in four random optical fields for each transwell, with three transwells per condition. Results are shown as percent invasion compared to invasion in the control cells. The *inset* shows p115RGS expression. Data is presented as mean ± SE from a single experiment that is representative of three independent experiments. * *p*<0.05 as determined by one-way ANOVA, followed by Tukey's multiple comparison test to obtain *p* values. (B) 4T1 cells were infected with adenovirus harboring GFP (control) or p115RGS, as indicated, for ∼18–19 h and starved for ∼9 h. Cells were then treated with 1 U/ml thrombin for 10 min, as indicated, before lysis in JNK activation buffer. Equal amounts of total protein in the lysates were separated by SDS-PAGE and immunoblot analysis was performed to assess levels of phospho-JNK (P-JNK, p54 and p46) and phospho-c-Jun (P-c-Jun). Total levels of JNK, myc-p115RGS and RhoGDI (loading control) are also shown. Data shown are from a single experiment that is representative of three independent experiments. (C) 4T1 cells were transfected with control (luc) and JNK siRNA for 67–68 h, and then starved for 8 h. Cells were allowed to invade Matrigel for 24 h in the presence of thrombin, towards 3% FBS. Cells that invaded were quantified as detailed for panel A. Results are shown as percent invasion compared to invasion in the control cells. The *inset* shows immunoblot analysis of JNK and Rho GDI (loading control) levels. Data is presented as mean ± SE from a single experiment that is representative of three independent experiments. ** *p*<0.01 as determined by one-way ANOVA, followed by Tukey's multiple comparison test.

Expression of p115RGS in the 4T1 mammary carcinoma cells led to a significant (∼75%) decrease in their ability to invade Matrigel in response to thrombin stimulation in transwell invasion assays (Figure 5A). Additionally, expression of p115RGS led to a decrease in Gα12QL-induced JNK as well as c-Jun phosphorylation in 4T1 cells (data not shown), suggesting that JNK is activated downstream of Rho. Hence, we determined if stimulation of 4T1 cells with thrombin activates JNK via G12 signaling. Treatment of 4T1 cells with thrombin did indeed lead to an increase in JNK as well as c-Jun phosphorylation (Figure 5B). Furthermore, expression of p115RGS in these cells significantly inhibited the phosphorylation of JNK and c-Jun in response to thrombin stimulation (Figure 5B). Finally, siRNA-mediated silencing of JNK expression significantly impaired stimulated invasion of 4T1 cells (Figure 5C). Taken together, our data indicates that JNK is activated downstream of receptor-mediated G12 signaling via Rho, and JNK activation is required for receptor-mediated 4T1 cell invasion.

## Discussion

In this paper, we demonstrate that activation of JNK is required for breast cancer cell invasion promoted by G12 signaling. We observe that Gα12-induced activation of JNK is accompanied by an increase in phosphorylation of c-Jun (a member of the AP-1 family of transcription factors) which is known to enhance its transcriptional activity and regulate the transcription of cell-invasion associated genes. In addition, we observe that JNK activation is required for thrombin-induced cell invasion, and JNK is likely activated downstream of G12 signaling via Rho since blocking G12 signaling by expression of p115RGS significantly decreases the level of JNK activation in 4T1 mammary carcinoma cells.

Activation of Rho is a critical component in the ability of G12 proteins to promote invasion of breast cancer cells [19]. In the current study, we demonstrate that JNK is activated downstream of Rho and ROCK since expression of dominant-negative RhoA or treatment of cells with the ROCK inhibitor significantly reduces the level of JNK phosphorylation induced by activated Gα12. ROCK inhibitor treatment also decreases Gα12-induced invasion, which would be expected if ROCK signals upstream of JNK. Activated Rho is known to regulate the transcription factor SRF via its ability to induce actin polymerization by a ROCK-LIMK-cofilin pathway [31], [37], and G12 signaling via Rho has been shown to lead to transcriptional activation of c-*fos* SRE and neoplastic transformation in fibroblasts [38]. Treatment of MDA-MB-231 cells with the ROCK inhibitor blocks G12-induced cell rounding and stress fiber formation (data not shown), whereas knockdown of JNK expression does not (Figure 4C). Therefore, G12 may be stimulating two parallel pathways downstream of ROCK, one regulating actin polymerization and the other leading to JNK activation, both culminating in AP-1 transcriptional activity to promote invasion of breast cancer cells.

Blockade of G12 signaling by p115RGS inhibits thrombin-stimulated invasion of MDA-MB-231 cells [19]. Interestingly, in the current study we found that p115RGS expression does not impact on thrombin-stimulated JNK activation in these cells (data not shown). Of note in this regard is that all G12-coupled receptors characterized thus far also activate one or more other G protein subfamilies, most notably Gq proteins [29]. Since the G12-coupled receptor PAR-1 (protease-activated receptor-1), which is activated by thrombin, has also been shown to stimulate Gq signaling, we determined the effect of blocking Gq signaling, by expression of RGS2, on JNK activation. RGS2 expression did not affect thrombin-induced JNK or c-Jun activation in either MDA-MB-231 cells or 4T1 cells (data not shown). Thus, while it appears that there are multiple pathways stimulated by thrombin that lead to JNK activation in MDA-MB-231 cells, thrombin-induced activation of JNK is predominantly mediated by G12 signaling in the 4T1 cells.

Cancer cell invasion is a complex phenomenon that involves interactions with the extracellular matrix and migration of cells. The changes in cytoskeletal dynamics required for cell migration are coordinated primarily by Rho GTPases [39], [40]. Interestingly, we observe a higher level of Gα12QL-activated Rho and increased stress fiber formation in JNK siRNA-treated cells versus control cells (Figure 4). This suggests the possible existence of a feedback pathway where by knocking down JNK expression leads to more Rho activation. Several studies have shown that the level of activated Rho appears to be critical during cell migration [41], [42], and in neutrophils excessive Rho activation mediated by constitutively-active Gα12/13 has been shown to inhibit polarization and motility [43]. In addition to regulating the level of activated Rho, JNK may be playing a role in the spatiotemporal control of Rho function to promote cell invasion. In this context, Gα13, and to a lesser extent Gα12, have been shown to interact with the JNK-interacting leucine zipper protein (JLP), a scaffolding protein involved in assembling the JNK signaling module including upstream MAPK kinase kinases and MAPK kinases that activate JNK and its downstream transcription factors [44]–[46]. It is interesting to note that microarray analysis of primary epithelial cell populations from nasopharyngeal biopsies showed an upregulation of several genes on the G12 signaling pathway including RhoA and JNK [47].

In conclusion, we have shown that JNK is an important player in the invasion of breast cancer cells promoted by G12 signaling, and that it is activated downstream of Rho and ROCK. The study also hints at an intriguing feedback loop between JNK activity and Rho activation. These findings further our understanding of the biology of breast cancer metastasis and implicate JNK as a potential therapeutic target.
